# High-frequency oscillations in scalp EEG lateralize to the epileptogenic hemisphere in children and adults

**DOI:** 10.1016/j.cnp.2025.09.004

**Published:** 2025-10-04

**Authors:** Dhruva P. Achar, Karunakar A. Kotegar, Kurupath Radhakrishnan

**Affiliations:** a Manipal Institute of Technology, Manipal Academy of Higher Education, Manipal, 576104, Karnataka, India; b Department of Computer Applications, Manipal University Jaipur, 303007, Rajasthan, India; c Department of Neurosciences, Avitis Institute of Medical Sciences, Nemmara, Palakkad, 678508, Kerala, India

**Keywords:** Scalp EEG, Epilepsy, Interictal epileptiform discharges, SOZ, HFOs, Seizure

## Abstract

**Objectives:**

High-frequency oscillations (HFO) in scalp electroencephalography (EEG) are promising biomarkers for localizing epileptogenic zones. Prior studies mainly used high sampling frequencies (>1000 Hz), whereas clinical monitoring often employs lower rates (<512 Hz). We assessed scalp EEG HFO detection feasibility at 512 Hz, examining whether HFO rates lateralize the epileptogenic hemisphere (EH) and localize seizure onset zone (SOZ).

**Methods:**

We retrospectively analyzed EEG from 32 patients during sleep, and seizures in 10 patients. A semi-automated algorithm combining band-pass filtering, sliding-window thresholding, and time-frequency validation was applied. HFO rates were compared between EH versus contralateral hemisphere (CH), and SOZ versus non-SOZ channels.

**Results:**

HFOs were detected in 27 patients, with higher rates in EH than CH (p = 0.0002). The asymmetry index lateralized EH in 22 patients (p = 0.0003). SOZ channels had higher HFO rates in 11 of 18 patients, though not significantly (p = 0.32). Pre-ictal HFO rates increased (p = 0.02), were higher in EH (p = 0.05), and decreased post-ictally (p = 0.004). Age-dependent decline was observed, with younger patients exhibiting higher median HFO rates than adults (≥18 years).

**Conclusions:**

Scalp EEG at 512 Hz reliably detects HFOs and lateralizes the EH, though SOZ localization remains limited.

**Significance:**

: Routine 512 Hz EEG can analyze HFOs, potentially enhancing presurgical evaluation.

## Highlights

• Scalp EEG at 512 Hz can reliably detect high-frequency oscillations (HFOs).
• HFO rates were significantly higher in the epileptogenic hemisphere than the contralateral hemisphere.
• Younger patients had higher median HFO rates, showing an age-dependent decline.

## 1 Introduction

Neurologists and neuroscientists frequently use scalp electroencephalography (EEG) to study brain functions and assess various neurological conditions. Traditionally, the frequency band of 0.5–70 Hz has been the primary focus of research, including in clinical settings. However, there is a growing interest in frequencies above 70 Hz. Frequencies greater than 80 Hz are referred to as high-frequency activity (HFA), while discrete EEG events characterized by at least four oscillations that clearly stand out from the background are known as high-frequency oscillations (HFOs) (Jacobs et al., 2009, Noorlag et al., 2019). HFOs can be further categorized into ripples (80–250 Hz) and fast ripples (250–500 Hz), and they can occur either physiologically or pathologically, spontaneously or as an evoked response (Thomschewski et al., 2019). Pathological HFOs have shown promise as biomarkers for identifying epileptogenic tissue in epilepsy, a disorder characterized by recurrent and unpredictable seizures, which pose significant challenges across various age groups (Fisher et al., 2014). Approximately 65 million people worldwide are affected by epilepsy, and despite ongoing advancements in antiseizure medications (ASMs), around 30% of patients experience drug-resistant epilepsy (DRE) (Kwan and Brodie, 2000). For this subgroup, epilepsy surgery presents a promising pathway to achieving seizure freedom. The primary goal of epilepsy surgery is to completely remove or disconnect the epileptogenic zone (EZ), the specific cortical area responsible for generating recurrent seizures (Lüders et al., 2006, Rosenow and Lüders, 2001). However, accurately identifying the location and extent of the EZ remains challenging due to the absence of a definitive marker that defines its boundaries (Tamilia et al., 2017). The EZ is a conceptual construct, and clinicians utilize various diagnostic tests to approximate its location (Rosenow and Lüders, 2001). During the initial phase of presurgical evaluation, patients undergo long-term video-electroencephalography monitoring (LTVEM) to capture electro-clinical features, including interictal (between seizures), pre-ictal (before seizure), ictal (during seizure), and post-ictal (after seizure) periods. These recordings assist epileptologists in identifying the seizure onset zone (SOZ), considered the best estimate of the EZ (Tamilia et al., 2017). However, this process is time-consuming (Kannan et al., 2021), and there is a risk of missing crucial episodes, which could hinder the achievement of anatomo-electro-clinical concordance, a vital criterion for considering surgery. This concordance refers to a well-defined brain magnetic resonance imaging (MRI) lesion that aligns with the seizure semiology and LTVEM data in a non-eloquent brain region. Interictal epileptiform discharges (IEDs) serve as another diagnostic tool, but their specificity is limited, as they have been observed in a small percentage of children without epileptic seizures (Borusiak et al., 2010). Even with LTVEM recordings, up to 20% of epilepsy patients may not exhibit IEDs (Werhahn et al., 2015), complicating the process of lateralizing the SOZ and further challenging the diagnostic pathway. Consequently, there is a clinical need for a reliable, objective, and specific biomarker to identify the hemisphere responsible for triggering seizures. Therefore, analyzing HFOs through scalp EEG could potentially benefit many subjects if it proves to be clinically valuable. Research on interictal HFOs using scalp EEG has shown promising results. Kobayashi et al. (2010) were the first to publish findings in 2010 using scalp EEG, revealing ripples occurring alongside epileptic spikes in children with epilepsy and continuous spike-waves during sleep (CSWS). Recent studies have identified scalp HFOs in both adults (Shi et al., 2024) and children (Cserpan et al., 2023, Cserpan et al., 2022b, Cserpan et al., 2022a, Nariai et al., 2020). Further research has shown that scalp HFOs are more specific and accurate than spikes and gamma frequencies in localizing the presumed EZ, though they may be less sensitive (Tamilia et al., 2020, Noorlag et al., 2022).

Most reported studies on HFOs have used intracranial EEG (iEEG) or scalp EEG recorded at sampling frequencies exceeding 1,000 Hz. However, in many busy epilepsy monitoring units (EMUs), LTVEM and routine video EEG are typically recorded at either 256 Hz or 512 Hz, as these lower sampling rates require significantly less data storage than rates exceeding 1,000 Hz. In clinical practice, epileptologists usually focus on detecting IEDs in EEG data filtered between 0.5 and 70 Hz to minimize high-frequency noise. In this context, sampling frequencies of 256 Hz or 512 Hz are sufficient for visualizing signals up to 70 Hz without aliasing, which is why detecting HFOs has not traditionally been a priority in EMUs. Nonetheless, exploring HFOs in scalp EEG recorded at 512 Hz could provide valuable insights. It would allow us to examine how frequently HFOs occur, whether they appear in a majority of patients, and if they are more prevalent in the epileptogenic hemisphere (EH) compared to the contralateral hemisphere (CH). Such insights could complement IED detection and potentially enhance presurgical evaluations. Additionally, while several scalp EEG studies have investigated HFO rates during sleep, few have analyzed HFO activity in the time frame just before seizure onset and immediately after seizure offset. Understanding how HFO rates fluctuate during these pre-ictal and post-ictal periods could offer critical information for predicting upcoming seizures and lateralizing seizure onset. In this study, we aimed to investigate HFO rates (80–250 Hz) using a semi-automated detection algorithm, in patients who underwent LTVEM at a sampling frequency of 512 Hz during sleep, as well as during the periods just before seizure onset and after seizure offset that occurred during sleep. Our primary objectives were to determine whether a higher HFO rate accurately identifies the EH and whether HFO rates in SOZ channels are higher than those in non-SOZ (NSOZ) channels. We also sought to examine the rate of HFO occurrences during pre-ictal and post-ictal periods to evaluate their potential role in seizure prediction and lateralizing the seizure onset.

## 2 Materials and methods

### 2.1 Patient details

We collected retrospectively the data of 32 patients with DRE with median age of 18 years (range: 3–32 years), who underwent LTVEM from September 2019 to July 2024 for electro-clinical characterization of their seizures or as presurgical evaluation at the Avitis Institute of Medical Sciences, Palakkad, Kerala, India. Their demographic and clinical features are summarized in Table 1. The ethical clearance for the study was obtained from the Institutional Ethics Committee, Kasturba Medical College, MAHE, Manipal (Project number is IEC1: 367/2022).

**Table 1:** Patient characteristics.

Patient No.	Sex	Age	Diagnosis	EH	SOZ (as in report)	Seizures during sleep
1	M	17	RTLE	Right	RT	Yes
2	F	24	RMTLE-HS	Right	RT	No
3	M	7	LHE	Left	LFC	No
4	M	17	PCE	Left	BPH, left more	Yes
5	F	18	PCE	Right	RPH	Yes
6	F	17	RIH	Left	LFC	Yes
7	F	23	LHS	Left	No SOZ	No
8	F	27	FLE	Right	RT	No
9	F	21	RPG	Right	RT	Yes
10	M	13	RIH	Left	LFC	Yes
11	F	28	LTLE	Left	LT	Yes
12	F	3	ROS	Right	No SOZ	No
13	F	17	AIE	Bilateral	BT	No
14	M	15	RTLE-AIE	Right	RT	Yes
15	F	7	NREMP	Right	No SOZ	No
16	M	16	PCE:Right	Right	RPH	No
17	M	21	LTS	Left	LFT	No
18	F	20	RPCE	Right	RPH	No
19	F	17	RTLE	Right	RT	No
20	M	28	DRFE	Left	LT	No
21	F	13	RMTLE-HS	Right	No SOZ	No
22	M	26	RMTLE-HS	Right	No SOZ	No
23	F	8	FE:RF	Right	No SOZ	No
24	M	6	LMTLE-HS	Left	No SOZ	No
25	M	32	LTLE	Left	No SOZ	No
26	M	31	LTLE	Left	No SOZ	No
27	M	9	SD	Right	RAH	Yes
28	F	22	PEE	Right	RT	No
29	M	32	LMTLE	Left	LT	No
30	M	18	RPCE	Right	RPH	No
31	M	23	RSG	Right	RFT	No
32	F	6	FCD	Right	RFC	Yes

Abbreviations: AIE — Autoimmune epilepsy, BPH — Bilateral posterior head, BT — Bilateral temporal, DRFE — Drug-resistant focal epilepsy, EH — Epileptogenic hemisphere, F — Female, FCD — Focal cortical dysplasia, FE:RF — Focal epilepsy: right frontal lesional, FLE — Frontal lobe epilepsy, LFC — Left fronto central, LFT — Left fronto temporal, LHE — Left hemispheric epilepsy, LHS — Left hemispheric seizures, LMTLE-HS — Left mesial temporal lobe epilepsy with hippocampal sclerosis, LT — Left temporal, LTLE — Left temporal lobe epilepsy, LTS — Left temporal plus syndrome, M — Male, NREMP — Non rapid eye movement parasomnia, No SOZ — No seizure onset zone mentioned in the report, PCE — Posterior cortex epilepsy, PEE — Post encephalitic epilepsy, RAH — Right anterior head, RFC — Right fronto central, RFT — Right fronto temporal, RIH — Right infantile hemiplegia, RPCE — Right posterior cortex epilepsy, RPH — Right posterior head, RMTLE-HS — Right mesial temporal lobe epilepsy with hippocampal sclerosis, RPG — Right parietal gliosis, RSG — Right subfrontal gliosis, RT — Right temporal, RTLE — Right temporal lobe epilepsy, SD — Seizure disorder, SOZ — Seizure onset zone, ROS — Recent onset seizure.

### 2.2 EEG recordings

All the patients underwent prolonged video-EEG recording for at least 9 h, except for patients #12, #14, #23 and #24 who underwent 1-hour routine video-EEG recording. The EEG was recorded using 19 channels with the sampling frequency of 512 Hz, with the electrode placement following 10–20 international standard. In patients #12, #14, #21, #24 and #25 the sampling frequency was 500 Hz. The acquired EEG of each patient was then exported to European data format (EDF) to perform quantitative analysis. During acquisition, the reference electrode was placed at Cz. After pre-processing, all EEG data were re-referenced to a bipolar montage for subsequent HFO detection and analysis.

### 2.3 Data selection

We selected a minimum of 20 min of non-rapid eye movement (NREM) sleep data that was free from significant artifacts. For the identification of NREM sleep periods, we divided the signal into consecutive, non-overlapping 30 s epochs and computed the ratio of delta band (1–4 Hz) power to total (1–40 Hz) power in each epoch. We then classified a recording as NREM if the mean delta ratio across all epochs exceeded 25% (Jiang et al., 2018, Li et al., 2021). For patients #3 and #27, the sleep duration considered exceeded 17 min. Additionally, for patients who experienced seizures during sleep, we selected 10 min of EEG data immediately preceding seizure onset and 5 min following seizure offset. This targeted approach was chosen to capture the temporal dynamics of HFO rates during the peri-ictal period encompassing the critical moments before and after seizures while minimizing the influence of interictal activity. This selection aligns with our objective of assessing the potential of HFOs as biomarkers for seizure prediction. Data from patients #17 and #18 were excluded from the analysis due to substantial power line noise interference in their EEG recordings, which remained dominant even after pre-processing, making them unsuitable for HFO analysis. We specifically chose sleep data because the likelihood of HFO occurrence is increased, while the presence of muscle artifacts and other noise is reduced during sleep compared to the awake period (Bagshaw et al., 2009, Clemens et al., 2003, Staba et al., 2004) . Details of the patients included in the analysis are shown in Table 1.

### 2.4 Pre-processing

**Fig. 1:**
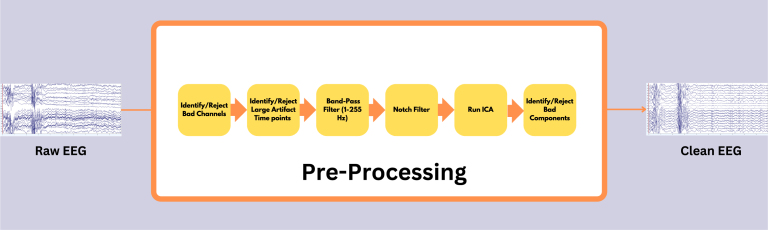
Pre-processing pipeline for cleaning raw EEG data. All pre-processing steps were performed using EEGLab (Delorme and Makeig, 2004).

**Fig. 2:**
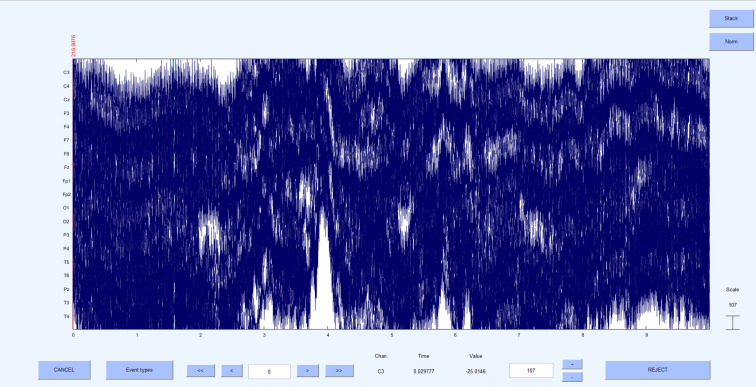
10 s of raw EEG data from a patient before applying the pre-processing pipeline. The EEG recordings exhibit high noise levels across all electrodes.

**Fig. 3:**
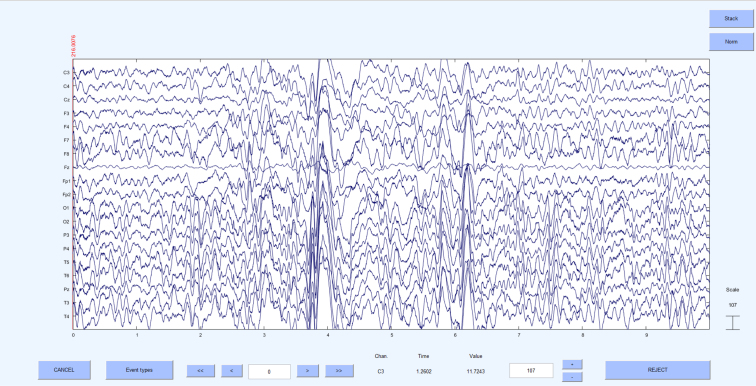
Pre-processed 10 s EEG signal obtained by applying the pre-processing pipeline to the raw EEG data shown in Fig. 2. The noise levels have been significantly reduced.

Fig. 1 illustrates the pre-processing pipeline applied before conducting HFO analysis. Initially, the data underwent a thorough review to identify electrodes affected by continuous noise throughout the recording duration; these electrodes were labeled as bad and excluded from further analysis. The signals were then scrutinized for large artifact time points across all electrodes, identified artifacts were deemed bad segments and excluded from further analysis. Next, the signals were band-pass filtered between 1 and 250 Hz using a high-order finite impulse response (FIR) filter (order = 3714, Kaiser window with β = 5.65), to eliminate slow drifts and high-frequency noise. To further reduce power line interference, a series of narrowband notch filters (2 Hz bandwidth) were applied at 50 Hz, 100 Hz, 150 Hz and 200 Hz to remove line noise and its harmonics. Subsequently, the signals underwent decomposition into independent components using the independent component analysis (ICA) infomax algorithm (Adib, 2012). The properties of each decomposed component, such as the power spectrum, time series, event-related potential (ERP) image, and power distribution across the scalp electrodes on the topomap, were reviewed to differentiate between artifactual components and brain components. Artifactual components were eliminated, and the retained components were reintegrated to generate a clean EEG signal. In some instances, artifact-related components, particularly those due to line noise, overlapped substantially with genuine neural signals. To avoid discarding valuable brain activity, we only removed components that were clearly dominated by artifacts. Any EEG data that continued to exhibit significant residual noise after this conservative cleaning approach was excluded from the final HFO analysis. Fig. 2 shows 10 s of raw EEG before the pre-processing stage, where noise levels in all the electrodes were very high. Fig. 3 shows the same 10 s segment after the pre-processing stage, demonstrating that the noises have been eliminated to produce a clean EEG. All the steps mentioned here were executed using the EEGLAB toolbox (Delorme and Makeig, 2004).

### 2.5 Analysis of scalp HFOs using a semi-automated approach

**Fig. 4:**
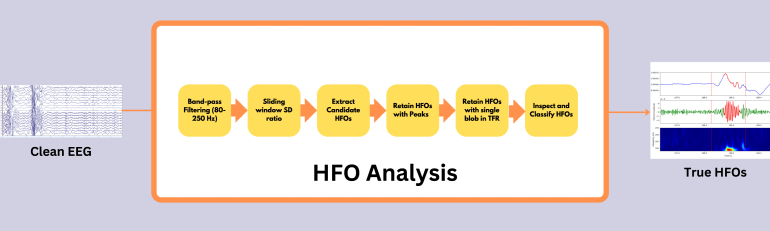
High-frequency oscillations (HFO) analysis pipeline as described in Section 2.5.

**Fig. 5:**
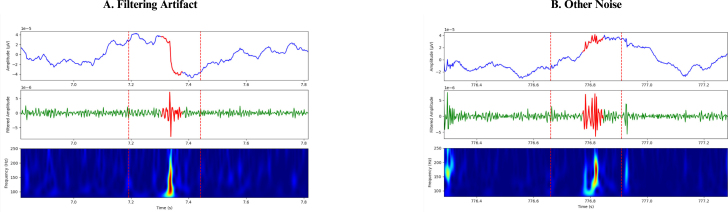
False high-frequency oscillations (HFO) seen from patient #1 sleep EEG, (A) HFO classified as filtering artifact during the visual verification of initially detected HFOs. (B) HFO classified as other noise during the visual verification of initially detected HFOs.

**Fig. 6:**
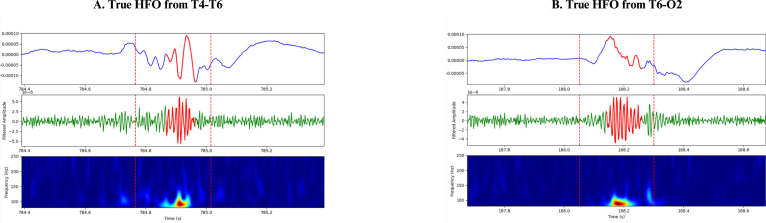
True high-frequency oscillations (HFO) seen from patient #1 sleep EEG who had right temporal lobe epilepsy (RTLE) and we observed high rate of HFOs in channels T4-T6 and T6-O2. (A) True HFO seen in channel T4-T6, (B) True HFO seen in channel T6-O2.

HFOs were defined as oscillatory events with a minimum of six cycles and a central frequency ranging between 80 and 250 Hz, clearly distinguishable from the background signal. The detection of HFOs was performed using a multi-step algorithm integrating signal processing, statistical analysis, and time–frequency analysis as shown in Fig. 4. First, the raw EEG signal X(t) was bandpass filtered to isolate the HFO frequency range (80–250Hz), yielding the filtered signal $X_{\mathrm{filtered}}\left(t\right)$. The rectified signal $X_{\mathrm{rectified}}\left(t\right)$ was then computed as the absolute value of the filtered signal:

(1) $$X_{\mathrm{rectified}}\left(t\right)=|X_{\mathrm{filtered}}\left(t\right)|$$

which was later used for amplitude-based validation. For initial detection, a 1000ms window was used as a baseline, and multiple 50ms windows with 50% overlap were slid within it. The standard deviation (SD) of $X_{\mathrm{filtered}}\left(t\right)$ was calculated in the 1000ms window, denoted $\sigma_{1000\mathrm{ms}}$, and in each 50ms window, denoted $\sigma_{50\mathrm{ms}}$. Their ratio,

(2) $$R=\frac{\sigma_{50\mathrm{ms}}}{\sigma_{1000\mathrm{ms}}}$$

was computed, and any 50ms window with R>1.7 (Li et al., 2024) was flagged. Consecutive flagged windows (between 1 and 7 in a row) were grouped into sequences, and each sequence’s center time $t_{\mathrm{center}}$ was used to extract a 250ms segment $\;\left(t_{\mathrm{center}}\pm 125\;\mathrm{ms}\right)$. These 250ms windows, considered candidate HFOs, were then subjected to amplitude-based validation. Within each segment, peaks in $X_{\mathrm{rectified}}\left(t\right)$ exceeding

(3) $$X_{\mathrm{rectified}}\left(t\right)\;>\;\mu_{\mathrm{rect}}\;+\;2\;\sigma_{\mathrm{rect}}$$

(where $\mu_{\mathrm{rect}}$ and $\sigma_{\mathrm{rect}}$ are the mean and SD of $X_{\mathrm{rectified}}\left(t\right)$ in a ±500ms window around $t_{\mathrm{center}}$) were counted. Candidate HFOs with fewer than six such peaks were discarded. Next, each candidate HFO passing the amplitude-based validation underwent time–frequency validation. The Stockwell transform was applied to compute the time–frequency representation (TFR) of $X_{\mathrm{filtered}}\left(t\right)$ over a 1s window centered at $t_{\mathrm{center}}$:

(4) $$\mathrm{TFR}\left(f,t\right)\;=\;\mathrm{Stockwell}\left[X_{\mathrm{filtered}}\left(t\right)\right]$$

and the same 250ms region $\;\left(t_{\mathrm{center}}\pm 125\;\mathrm{ms}\right)$ was extracted from this TFR. Points in the 250ms TFR segment that exceeded

(5) $$\mathrm{TFR}\left(f,t\right)\;>\;\mu_{\mathrm{TFR}}\;+\;2\;\sigma_{\mathrm{TFR}}$$

(where $\mu_{\mathrm{TFR}}$ and $\sigma_{\mathrm{TFR}}$ are the mean and SD of the TFR values over the 1s window) were identified and subjected to connected-component labeling. Candidate HFOs displaying exactly one connected blob were classified as confirmed HFOs. This detection procedure was repeated independently for each EEG channel, and the resulting HFOs were later visually validated to distinguish between true and false events. The identified HFOs then underwent further visual confirmation to exclude false-positive events such as ringing (filtering sharp transients) (Bénar et al., 2010), muscle artifacts, and electrode artifacts. Fig. 5, Fig. 6 illustrate instances of HFOs visually classified as false and true HFOs from patient #1’s sleep EEG.

**Fig. 7:**
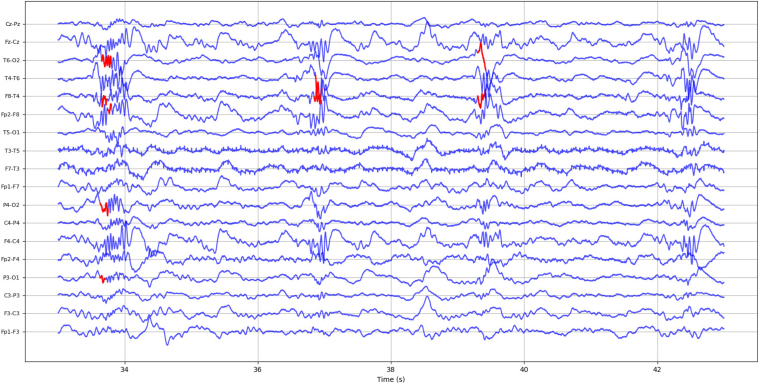
10 s of EEG segment from patient #1 sleep EEG showing true retained high-frequency oscillations (HFO) marked in red, as can be seen the rate of HFOs were more in right temporal chain (RTC) as the patient had right temporal lobe epilepsy (RTLE).

Each of the 18 channels “Fp1-F3, F3-C3, C3-P3, P3-O1” - left parasagittal chain (LPC); “Fp2-F4, F4-C4, C4-P4, P4-O2” - right parasagittal chain (RPC); “Fp1-F7, F7-T3, T3-T5, T5-O1” – left temporal chain (LTC); “Fp2-F8, F8-T4, T4-T6, T6-O2” - right temporal chain (RTC); ”Fz-Cz, Cz-Pz” - midline chain (MC) was assessed to record the number of confirmed HFO events. In the case of patient #2’s sleep EEG, only 17 channels were considered due to the removal of channel Fz during the pre-processing stage. Fig. 7 illustrates the automatically detected and visually confirmed HFOs for patient #1’s sleep EEG.

The total HFO rate (HFO/min) of each channel was summed to provide the HFO rate for each region in each patient as follows:

(6) $$\text{HFORate(LPC)}=\text{HFORate}\left[\text{(Fp1-F3)}+\text{(F3-C3)}+\text{(C3-P3)}+\text{(P3-O1)}\right]$$

(7) $$\text{HFORate(LTC)}=\text{HFORate}\left[\text{(Fp1-F7)}+\text{(F7-T3)}+\text{(T3-T5)}+\text{(T5-O1)}\right]$$

(8) $$\text{HFORate(MC)}=\text{HFORate}\left[\text{(Fz-Cz)}+\text{(Cz-Pz)}\right]$$

(9) $$\text{HFORate(RPC)}=\text{HFORate}\left[\text{(Fp2-F4)}+\text{(F4-C4)}+\text{(C4-P4)}+\text{(P4-O2)}\right]$$

(10) $$\text{HFORate(RTC)}=\text{HFORate}\left[\text{(Fp2-F8)}+\text{(F8-T4)}+\text{(T4-T6)}+\text{(T6-O2)}\right]$$

Similarly, the HFO rates for the right hemisphere (RH) and left hemisphere (LH) were obtained as follows:

(11) $${\text{HFORate}}_{\text{Right}}=\text{HFORate}\left[\text{RTC}+\text{RPC}+\text{MC}\right]$$

(12) $${\text{HFORate}}_{\text{Left}}=\text{HFORate}\left[\text{LTC}+\text{LPC}+\text{MC}\right]$$

### 2.6 Asymmetry in scalp EEG HFO rate relative to the EH

The asymmetry index (AI) was computed to quantify the lateralization of HFO rates between hemispheres. The AI is defined by the following formula:

(13) $$\text{AI}=\frac{{\text{HFORate}}_{\text{Right}}-{\text{HFORate}}_{\text{Left}}}{{\text{HFORate}}_{\text{Right}}+{\text{HFORate}}_{\text{Left}}}$$

The resulting values range from −1 (indicating HFO rates are disproportionately larger in the LH) to ＋1 (indicating HFO rates are disproportionately larger in the RH) (Nariai et al., 2020). After calculating the AI for each patient, we compared the result to the clinically determined EH. Concordant lateralization was defined as a positive AI value aligning with a right-sided EH or a negative AI aligning with a left-sided EH. This approach enabled a quantitative evaluation of whether the HFO rates consistently lateralized to the hemisphere identified as epileptogenic by clinical assessment.

### 2.7 Statistical analysis

Statistical analyses were conducted using Python, primarily utilizing the pandas (Wes McKinney, 2010) and scipy (Virtanen et al., 2020) libraries. To determine whether HFO rates differed significantly between the EH and the CH, we performed a Wilcoxon signed-rank test. This non-parametric test was selected because the data were continuous, involved matched pairs, and did not meet the normality assumption. The Wilcoxon signed-rank test evaluates differences in the median HFO rates between groups. We also investigated whether HFO rates differed between the SOZ and the NSOZ channels for patients who experienced seizures during their recordings and whose SOZ was identified in the report. Again, a Wilcoxon signed-rank test was used to compare HFO rates between SOZ and NSOZ channels. Furthermore, we employed the Wilcoxon signed-rank test to assess whether HFO rates were significantly different between the pre-ictal and post-ictal periods. For the AI values, we evaluated whether the index value in the majority of the patients correctly lateralized the EH. We also examined whether high HFO rates accurately identified the SOZ in most patients. Since the variable of interest was binary and the observations were independent, we used a binomial test to determine the statistical significance of the proportion of correct identifications. To visually represent our find ings and further validate our statistical results, we created box plots and swarm plots using the seaborn library (Waskom, 2021). The box plots illustrated the distribution of HFO rates, while the swarm plots overlaid individual data points on top of the box plots. P-values from the Wilcoxon signed-rank tests and the binomial test were annotated on the plots to indicate statistical significance.

## 3 Results

### 3.1 Patient characteristics

Out of the 30 patients considered for analysis, 10 had seizures during sleep. The SOZ was not reported for patients #7, #12,#15 and #21-26; therefore, they were excluded from the comparison of HFO rates between SOZ and NSOZ channels. Table 2 shows the channels identified as SOZ channels in patients whose reports included the SOZ. Patients #4 and #13 had bilateral onset. In patient #4, out of 16 electro-clinical seizures recorded, 13 manifested in the left posterior head (LPH) region; therefore, we considered the LH to be the dominant hemisphere. Patient #13 had 7 electro-clinical seizures, with 4 having a right temporal (RT) onset and 3 having a left temporal (LT) onset. The dominant hemisphere was not mentioned in the report; therefore, patient #13 was excluded from the comparison of HFO rates between the EH and CH.

**Table 2:** The seizure onset zone (SOZ) channels for each patient whose report had SOZ mentioned. The SOZ region was determined by grouping the channels closest to the region specified as the SOZ in the patient’s report.

Patient No.	SOZ (as in report)	SOZ Channels
1	RT	Fp2-F8, F8-T4, T4-T6, T6-O2
2	RT	Fp2-F8, F8-T4, T4-T6, T6-O2
3	LFC	Fp1-F3, F3-C3, C3-P3, Fz-Cz, Cz-Pz
4	BPH, Left more	T3-T5, T5-O1, C3-P3, P3-O1, Cz-Pz
5	RPH	T4-T6, T6-O2, C4-P4, P4-O2, Cz-Pz
6	LT	Fp1-F7, F7-T3, T3-T5, T5-O1
9	RT	Fp2-F8, F8-T4, T4-T6, T6-O2
10	LFC	Fp1-F3, F3-C3, C3-P3, Fz-Cz, Cz-Pz
11	LT	Fp1-F7, F7-T3, T3-T5, T5-O1
13	BT	Fp1-F7, F7-T3, T3-T5, T5-O1, Fp2-F8, F8-T4, T4-T6, T6-O2
14	RT	Fp2-F8, F8-T4, T4-T6, T6-O2
16	RPH	T4-T6, T6-O2, C4-P4, P4-O2, Cz-Pz
19	RT	Fp2-F8, F8-T4, T4-T6, T6-O2
20	LT	Fp1-F7, F7-T3, T3-T5, T5-O1
27	RAH	Fp2-F8, F8-T4, Fp2-F4, F4-C4, Fz-Cz
28	RT	Fp2-F8, F8-T4, T4-T6, T6-O2
30	RPH	T4-T6, T6-O2, C4-P4, P4-O2, Cz-Pz
32	RFC	Fp2-F4, F4-C4, C4-P4, Fz-Cz, Cz-Pz

Abbreviations: BPH — Bilateral posterior head, BT — Bilateral temporal, LFC — Left fronto central, LT — Left temporal, RAH — Right anterior head, RFC — Right fronto central, RPH — Right posterior head, RT — Right temporal.

### 3.2 HFO rate during sleep

**Fig. 8:**
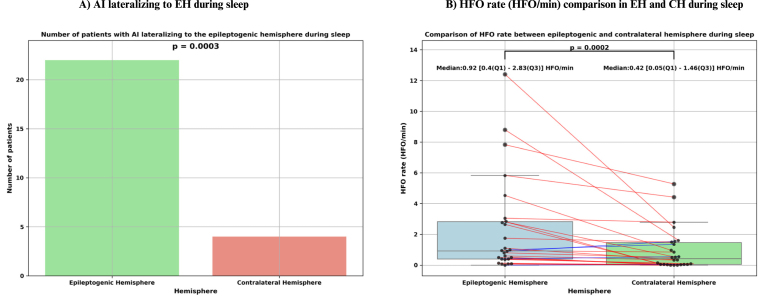
Statistical analysis of high-frequency oscillations (HFO) rate (HFO/min) during sleep. (A) Bar plot illustrating the asymmetry index (AI), demonstrating significant lateralization of HFO rate to the epileptogenic hemisphere (EH) during sleep (p = 0.0003). (B) Box plot comparing HFO rate between the EH and the contralateral hemisphere (CH). Each dot represents data from an individual patient. Paired values from the same patient are connected by lines, with red lines indicating higher HFO rate in the EH and blue lines indicating higher rate in the CH. Median and interquartile range ([Q1–Q3]) values are provided for each group. HFO rates were significantly higher in the EH (median: 0.92 [0.40–2.83] HFO/min) compared to the CH (median: 0.42 [0.05–1.46] HFO/min;p = 0.0002).

Out of the 30 patients whose sleep data were analyzed, HFOs were detected in 27 of them. The total analyzed sleep time across these patients was 659 min and 28 s. The HFO detection algorithm identified 4,035 HFOs in total, of which 1,663 were retained after visual verification. With the exception of patients #12, #14, #15, and #22 where HFO rates were higher in the CH than in the EH, all other patients exhibited a higher HFO rate in the EH compared to the CH. The AI values lateralized to the EH in 22 out of 26 patients, achieving statistical significance (p = 0.0003, p<0.0005) (see Fig. 8). Additionally, HFO rates in the EH were significantly greater (p = 0.0002, p<0.0005), with a median HFO rate in the EH of 0.92 [0.4–2.83] HFO/min compared to 0.42 [0.05–1.46] HFO/min in the CH (see Fig. 8).

**Fig. 9:**
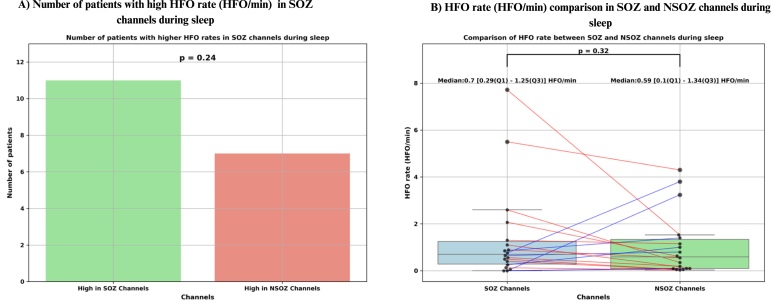
Statistical analysis of high-frequency oscillations (HFO) rate (HFO/min) during sleep. (A) Bar plot showing the number of patients (out of 18) who had higher HFO rate in seizure onset zone (SOZ) channels compared to non-SOZ (NSOZ) channels. Although 11 patients exhibited higher HFO rate in SOZ channels, this difference was not statistically significant (p = 0.24). (B) Box plot comparing HFO rate between SOZ and NSOZ channels. Each dot represents data from a single patient, with paired values connected by lines. Red lines indicate higher HFO rate in SOZ channels, and blue lines indicate higher rate in NSOZ channels. Median and interquartile range ([Q1–Q3]) values are annotated for each group. HFO rates were not significantly different between SOZ (median: 0.70 [0.29–1.25] HFO/min) and NSOZ channels (median: 0.59 [0.10–1.34] HFO/min;p = 0.32).

Among the 27 patients with detected HFOs, the SOZ was specified in the reports of 18 patients. In 11 of these cases, a higher HFO rate in the SOZ channels correctly identified the SOZ; however, this finding did not reach statistical significance (p = 0.24) (see Fig. 9). Furthermore, the HFO rate in the SOZ channels was not significantly greater than the NSOZ channels (p = 0.32), with a median HFO rate of 0.7 [0.29–1.25] HFO/min in the SOZ channels compared to 0.59 [0.1–1.34] HFO/min in the NSOZ channels (see Fig. 9). Table 3 shows the HFO rate and AI values seen during sleep.

**Table 3:** High-frequency oscillations (HFO) rate and asymmetry index (AI) during sleep.

Patient No.	HFO rate (HFO/min)	AI	HFO rate (HFO/min) in SOZ channels	HFO rate (HFO/min) in NSOZ channels
1	2.95	0.90	2.60	0.35
2	2.69	0.96	2.07	0.63
3	9.25	−0.19	7.72	1.53
4	4.57	−0.04	0.76	3.81
5	0.74	0.30	0.56	0.19
6	1.25	−0.23	0.25	1.00
7	0.50	−1.00	No SOZ	
9	9.80	0.69	5.50	4.30
10	1.45	−0.06	0.65	0.80
11	0.09	−1.00	0.00	0.09
12	2.30	−0.27	No SOZ	
13	1.20	−0.68	1.10	0.10
14	2.25	−0.15	0.85	1.40
15	0.90	−0.16	No SOZ	
16	0.55	0.82	0.50	0.05
19	0.10	0.33	0.00	0.10
20	0.17	−0.60	0.13	0.03
21	5.41	0.65	No SOZ	
22	0.05	−1.00	No SOZ	
23	0.46	0.60	No SOZ	
24	9.58	−0.14	No SOZ	
25	14.86	−0.67	No SOZ	
26	0.083	−1.00	No SOZ	
27	3.29	0.68	0.06	3.24
28	1.44	0.53	0.89	0.55
30	0.45	0.78	0.40	0.05
32	2.45	0.08	1.30	1.15
**Summary** (**Median [Q1–Q3]**) **HFO/min**	**1.44 [0.48–3.12]**	–	**0.7 [0.29–1.25]**	**0.59 [0.1–1.34]**

Note: HFOs were not detected in patients #8, #29, and #31. No SOZ — No seizure onset zone was mentioned in the reports for patients #7, #12, #15, and #21-26. As a result, these patients were excluded from the comparison of HFO rates between SOZ and non-SOZ (NSOZ) channels. Bilateral — Additionally, patient #13 had bilateral seizure onset, leading to the exclusion from the comparison of HFO rates between the epileptogenic hemisphere (EH) and the contralateral hemisphere (CH).

### 3.3 HFO rate before and after seizure

**Fig. 10:**
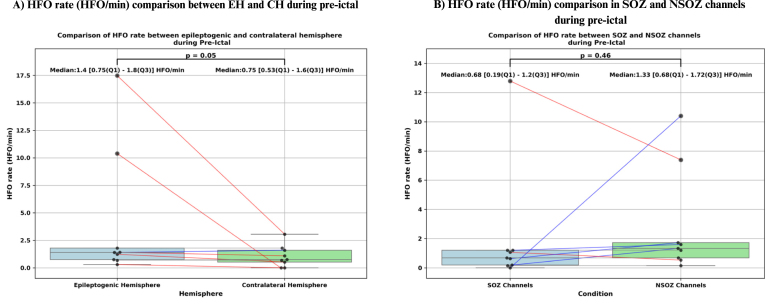
Statistical analysis of high-frequency oscillations (HFO) rate (HFO/min) during pre-ictal. (A) Box plot comparing HFO rate between the epileptogenic hemisphere (EH) and the contralateral hemisphere (CH). Each dot represents an individual patient, and paired values are connected by lines. Red lines indicate higher HFO rate in the EH, and blue lines indicate higher rate in the CH. Median [Q1–Q3] HFO rates were higher in the EH (1.40 [0.75–1.80] HFO/min) compared to the CH (0.75 [0.53–1.60] HFO/min), and this difference was statistically significant (p = 0.05). (B) Box plot comparing HFO rate between seizure onset zone (SOZ) and non-SOZ (NSOZ) channels. Each dot represents data from an individual patient, with red lines indicating higher rate in SOZ channels and blue lines indicating higher rate in NSOZ channels. Median [Q1–Q3] HFO rates were 0.68 [0.19–1.20] HFO/min in SOZ channels and 1.33 [0.68–1.72] HFO/min in NSOZ channels. However, this difference was not statistically significant (p = 0.46).

**Fig. 11:**
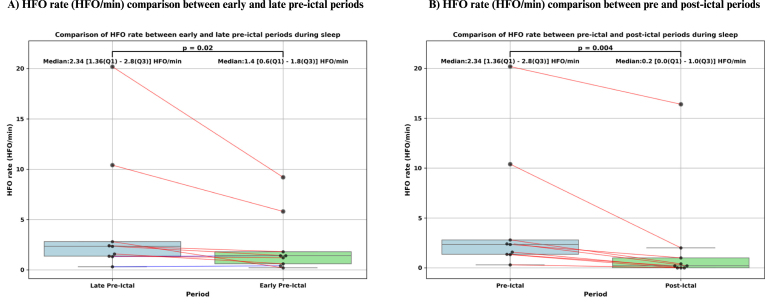
Statistical analysis of high-frequency oscillations (HFO) rate (HFO/min) during pre-ictal and post-ictal. (A) Box plot comparing HFO rate between the early (10–5 min before seizure onset) and late (5–0 min before seizure onset) pre-ictal periods. Each dot represents an individual patient, with red lines indicating a higher HFO rate in the late period and blue lines indicating a higher rate in the early period. HFO rates significantly increased closer to seizure onset (p = 0.02), with median [Q1–Q3] rates of 2.34 [1.36–2.80] HFO/min in the late period and 1.40 [0.60–1.80] HFO/min in the early period. (B) Box plot comparing HFO rate during the 5 min period before seizure onset (pre-ictal) and the 5 min period after seizure offset (post-ictal). Paired values are connected with red lines when the pre-ictal HFO rate is higher and blue lines when the post-ictal HFO rate is higher. A significant decrease in HFO activity was observed post-ictally (p = 0.004), with median [Q1–Q3] rates of 2.34 [1.36–2.80] HFO/min pre-ictally and 0.20 [0.00–1.00] HFO/min post-ictally.

Out of the 10 patients analyzed, we examined data from 10 min before seizure onset to 5 min after seizure offset. HFOs were detected in 9 patients before seizure onset and in 6 patients after seizure offset. The total pre-ictal time analyzed across the 10 patients was 100 min. The HFO detection algorithm identified a total of 1,050 HFOs, of which 335 were retained after visual verification. The total post-ictal time analyzed for the same patients was 50 min, during which the algorithm detected 379 HFOs, with 101 retained after verification. During the pre-ictal period, the rate of HFOs was significantly higher in the 5 min immediately before seizure onset (p = 0.02, p<0.05), with a median HFO rate of 2.34 [1.36–2.8] HFO/min (see Fig. 11). Additionally, during this period, the HFO rate was significantly higher in the EH (p = 0.05,p = 0.05), with a median of 1.4 [0.75–1.8] HFO/min, compared to 0.75 [0.53–1.6] HFO/min in the CH (see Fig. 10). However, we did not observe a significant difference in HFO rates between SOZ channels (median: 0.68 [0.19–1.2] HFO/min) and NSOZ channels (median: 1.33 [0.68–1.72] HFO/min) during the same period (p = 0.46) (see Fig. 10).

**Fig. 12:**
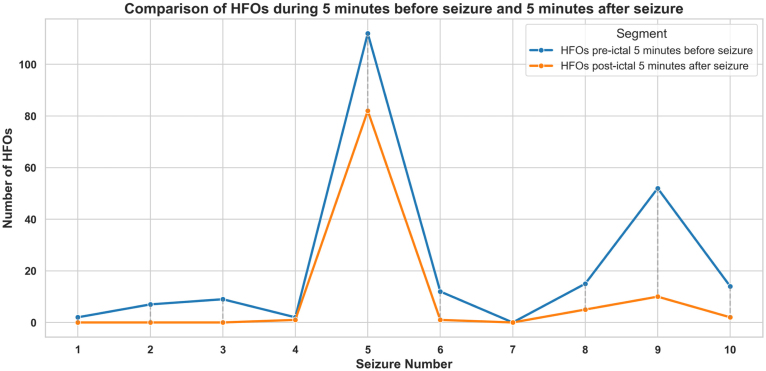
Comparison of the number of high-frequency oscillations (HFO) that occurred during the 5 min before seizure onset and the 5 min after seizure offset. Except for seizure number 7 from patient #11, which showed no HFOs detected in either the pre-ictal or post-ictal periods, all other seizures in the 9 patients demonstrated a higher number of HFOs in the 5 min preceding seizure onset compared to the 5 min following seizure offset.

Furthermore, the HFO rates detected in the 5 min before seizure onset, with a median of 2.34 [1.36–2.8] HFO/min, were significantly higher than those in the 5 min following seizure offset, which had a median of 0.2 [0.0–1.0] HFO/min (p = 0.004, p<0.005) (see Fig. 11). Fig. 12 presents a graphical comparison of the number of HFOs observed in the 5 min before seizure onset and the 5 min after seizure offset. Table 4 presents the HFO rate and AI values during the 10 min before the seizure onset and 5 min after seizure offset.

**Table 4:** High-frequency oscillations (HFO) rate and asymmetry index (AI) during 10 min pre-ictal and 5 min post-ictal periods.

Patient No.	Pre-Ictal				Post-Ictal			
	HFO rate (HFO/min)	AI	HFO rate (HFO/min) in SOZ channels	HFO rate (HFO/min) in NSOZ channels	HFO rate (HFO/min)	AI	HFO rate (HFO/min) in SOZ channels	HFO rate (HFO/min) in NSOZ channels
1	0.70	1.00	0.55	0.15	0.00	0.00	0.00	0.00
4	2.72	0.07	0.39	2.53	0.00	0.00	0.00	0.00
5	2.18	0.55	1.46	0.73	0.00	0.00	0.00	0.00
6	2.56	−0.36	1.08	1.48	0.20	−1.00	0.20	0.00
9	29.38	0.72	19.19	10.19	16.40	0.69	5.00	11.40
10	4.20	0.33	1.40	2.80	0.20	1.00	0.00	0.20
14	3.74	−0.07	0.63	3.12	1.00	0.20	0.20	0.80
27	16.20	1.00	0.00	16.20	2.00	0.80	0.20	1.80
32	3.00	0.05	1.20	1.80	0.40	0.00	0.00	0.40
**Summary** (**Median [Q1–Q3]**) **HFO/min**	**3.0 [2.56–4.2]**	–	**1.08 [0.55–1.4]**	**2.53 [1.48–3.12]**	**0.2 [0.0–1.0]**	–	**0.0 [0.0–0.2]**	**0.2 [0.0–0.8]**

Note: No SOZ — No seizure onset zone. HFOs were not detected in patient #11 during both the pre-ictal and post-ictal periods. In patients #1, #4, and #5, HFOs were detected only in the pre-ictal period and not during the post-ictal period.

### 3.4 HFO rate and age

**Fig. 13:**
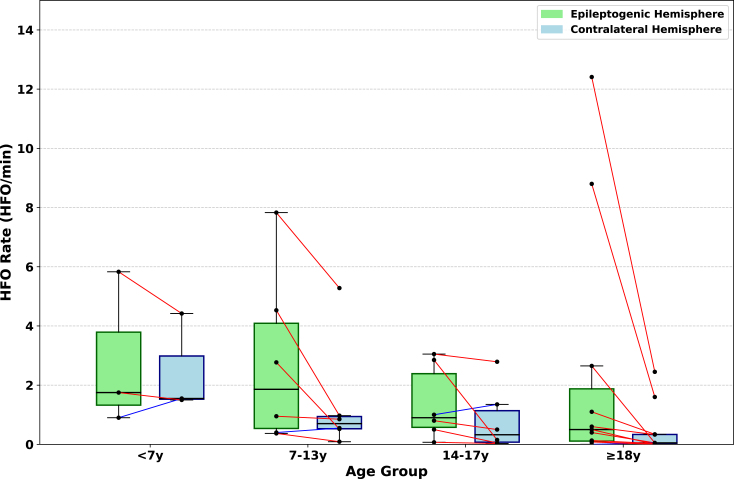
Comparison of high-frequency oscillation (HFO) rates across age groups. Box plot comparing the HFO rates (HFO/min) in the epileptogenic hemisphere (EH, green) and contralateral hemisphere (CH, blue), stratified into four age groups: children <7 years, children 7–13 years, adolescents 14–17 years, and adults (≥18 years). Median HFO rates were highest in younger children and progressively declined with age. Specifically, EH rates were 1.75 [1.32–3.79] HFO/min (<7 years), 1.86 [0.54–4.09] HFO/min (7–13 years), 0.90 [0.57–2.39] HFO/min (14–17 years), and 0.50 [0.11–1.88] HFO/min (≥18 years). CH rates were 1.55 [1.52–2.98] HFO/min (<7 years), 0.70 [0.53–0.94] HFO/min (7–13 years), 0.32 [0.08–1.14] HFO/min (14–17 years), and 0.05 [0.02–0.34] HFO/min (≥18 years). Each dot represents an individual patient, with red lines indicating higher HFO rates in the EH and blue lines indicating higher HFO rates in the CH within matched pairs.

Our analysis demonstrates a clear relationship between age and HFO rate (HFO/min), consistent with previous literature. When patients were stratified into four age groups, children younger than 7 years (n = 3; ages: 3, 6, 6), children aged 7–13 years (n = 6; ages: 7, 7, 8, 9, 13, 13), adolescents aged 14–17 years (n = 6 ; ages: 15, 16, 17, 17, 17, 17), and adults aged 18 years or older (n = 11; ages: 18, 18, 21, 22, 23, 24, 26, 28, 28, 31, 32), we found that younger patients exhibited higher median HFO rates, with a progressive decline across the older age groups (see Fig. 13).

In the EH, the median HFO rate for children younger than 7 years was 1.75 [1.32–3.79] HFO/min. For children aged 7 to 13 years, the median HFO rate was 1.86 [0.54–4.09] HFO/min. Among adolescents aged 14 to 17 years, the median rate was 0.90 [0.57–2.39] HFO/min, while adults aged 18 years or older had a median HFO rate of 0.50 [0.11–1.88] HFO/min.

A similar age-dependent decline was observed in the CH. The median HFO rate for children younger than 7 years was 1.55 [1.52–2.98] HFO/min. In the group aged 7 to 13 years, the median rate was 0.70 [0.53–0.94] HFO/min. Adolescents aged 14 to 17 years displayed a median rate of 0.32 [0.08–1.14] HFO/min, and adults aged 18 years or older had the lowest median HFO rate at 0.05 [0.02–0.34] HFO/min. These findings are in line with previous studies reporting that scalp HFOs are more frequent and more readily detected in young children, with a progressive decrease as age increases (Cserpan et al., 2021, Windhager et al., 2022). This age-dependent decline is believed to reflect not only developmental changes in brain excitability and epileptiform activity, but also biophysical factors such as increased skull thickness and changes in skull conductivity, both of which act as low-pass filters and attenuate high-frequency signals at the scalp (Wendel et al., 2010). However, studies have since demonstrated that, with appropriate recording parameters and rigorous analysis, it is indeed possible to capture these events noninvasively (Andrade-Valenca et al., 2011). Subsequent work has consistently confirmed that fast oscillations in the gamma (40–80 Hz) and ripple (> 80 Hz) bands can be reliably recorded from the scalp in adult patients, reinforcing their potential as a noninvasive biomarker (Melani et al., 2013).

We are confident in the validity of the HFOs detected in our adult patients for several reasons. First, our findings demonstrate clear lateralization value. The HFO rate in adults was higher in the EH (0.50 [0.11–1.88] HFO/min) than in the CH (0.05 [0.02–0.34] HFO/min), indicating a strong association with the presumed source of epileptic activity. This is consistent with studies showing that scalp HFOs, especially ripples, serve as highly specific and accurate markers for the SOZ, in some cases outperforming traditional interictal spikes (Andrade-Valenca et al., 2011, Melani et al., 2013). Secondly, the observed HFOs were more in the regions where the IEDs were found which provides further evidence of their pathological origin. Finally, our analysis included careful visual inspection to distinguish genuine cerebral HFOs from artifacts, such as irregular muscle activity, a crucial step in ensuring the validity of scalp-recorded HFOs.

### 3.5 Case representation

**Fig. 14:**
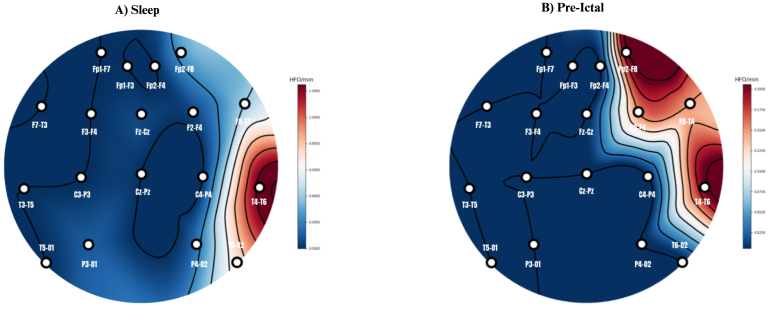
Scalp map showing the high-frequency oscillations (HFO) rate (HFO/min) in patient #1 during (A) Sleep, and (B) Pre-ictal. Patient #1 experienced 4 electro-clinical seizures, with semiology and ictal EEG favoring a right temporal (RT) onset. The image shows high HFO rates in the RT region during both sleep and pre-ictal periods. No HFOs was seen during post-ictal period.

Patient #1, with DRE, underwent LTVEM as part of a presurgical evaluation. This monitoring revealed frequent IEDs in the RT region and the 4 electro-clinical seizures that occurred during the recording consistently originated from the RT region, confirming the localization of SOZ in the right temporal lobe (RTL). The patient subsequently had surgery that included a right anterior temporal lobectomy with posterior temporo-occipital disconnection. Intraoperative electrocorticography (ECoG) confirmed the presence of numerous spikes in the right posterior temporal (RPT) and temporo-occipital regions. These spikes were completely eliminated following resection, indicating successful removal of the spiking zone. Follow-up EEGs conducted at 6 days, 3 months, and 1 year post-surgery showed no recorded electro-graphic or electro-clinical seizures, with only rare RPT sharp waves observed at the 1-year mark. Clinically, the patient remained seizure-free. Analysis of HFOs in the patient’s presurgical scalp EEG data revealed significantly higher HFO rates in the RT channels (T4–T6, T6–O2, F8–T4, and Fp2–F8) where IEDs and seizure activity had previously been identified. Fig. 14 illustrates the scalp maps of HFO rates during both sleep and pre-ictal periods, further supporting the localization of the SOZ. This retrospective finding highlights the potential of HFO analysis as an additional biomarker for seizure lateralization. It suggests that incorporating HFO analysis into presurgical evaluations could enhance the precision of SOZ localization and improve overall presurgical assessment.

## 4 Discussion

This study examined the feasibility and clinical relevance of detecting HFOs in scalp EEG data collected during LTVEM at a sampling frequency of 512 Hz. We specifically explored whether HFO rates could help in lateralizing the EH and localize the SOZ. Additionally, we examined the variation of the HFO rates during the pre-ictal and the post-ictal periods.

### 4.1 Feasibility of HFO detection at 512 Hz

Our semi-automated pipeline successfully identified HFOs in the range of 80–250 Hz in most patients. This finding suggests that HFOs can be effectively captured and analyzed at a sampling frequency of 512 Hz, or in some cases, 500 Hz. This is significant because many clinical EEG recordings are traditionally collected at a 512 Hz sampling rate due to data storage constraints. Previous studies on scalp HFOs often utilized higher sampling rates (greater than 1000 Hz), which may not always be feasible in a busy EMU. By demonstrating that meaningful HFO analysis can be conducted at a lower sampling frequency, our results enhance the practical applicability of HFO-based biomarkers in routine clinical practice.

### 4.2 Lateralizing value of HFOs

HFOs were detected in the majority of patients, and in most cases, the rate of HFOs was significantly higher in the EH compared to the CH. This observed lateralization, as reflected in the AI values, reached statistical significance, suggesting that HFO rates may serve as an objective marker for identifying the hemisphere responsible for seizure generation. This finding is clinically important because determining hemisphere lateralization is a crucial step in presurgical evaluation, which can help guide further, more localized investigations.

### 4.3 Localizing the SOZ

Although HFO rates were significantly higher in the EH, we did not observe a statistically significant difference between HFO rates in SOZ channels and NSOZ channels. Several factors may explain this finding. First, scalp EEG recordings have lower spatial resolution compared to iEEG; meaning that HFOs generated at a localized cortical site may be detected by neighboring scalp electrodes due to volume conduction, which could dilute the specificity for identifying the SOZ. Second, the relatively small sample of patients with a clearly defined SOZ may have limited the statistical power to detect differences. Thus, while scalp HFOs appear promising for hemisphere level lateralization, their ability to accurately identify the precise SOZ remains uncertain in this study.

### 4.4 HFO rates in the pre-ictal and post-ictal periods

Our analysis of HFO rates during the 10 min pre-ictal and 5 min post-ictal periods revealed significant temporal variations. We found that the HFO rate was significantly higher in the 5 min immediately preceding seizure onset, suggesting a potential role of HFOs as pre-ictal biomarkers. This pre-ictal surge in HFO activity suggests that these oscillations could be integral not only to the lateralization of the EH but also for predicting the upcoming seizures. The ability to detect such changes in scalp EEG could contribute to seizure prediction algorithms and real-time monitoring applications. In contrast, the post-ictal period exhibited a significant decrease in HFO rates, with far fewer HFOs occurring in the 5 min after seizure offset compared to the pre-ictal period. Understanding these post-ictal dynamics could aid in refining seizure prediction models and help differentiate between pathological and physiological HFOs. Although these findings are preliminary, they underscore the potential of scalp HFO analysis as a real-time tool for seizure forecasting and for enhancing our understanding of peri-ictal network dynamics.

### 4.5 Clinical implications and future directions

From a clinical perspective, our results suggest that integrating HFO analysis into conventional LTVEM pipelines, even with a 512 Hz sampling rate could enhance the evaluation of patients with DRE. If used alongside established metrics such as IEDs and seizure semiology, HFO rates may facilitate more rapid lateralization, thereby supporting the presurgical planning. Furthermore, the observed increase in pre-ictal HFO rates suggests a possible application in seizure forecasting, which could benefit patients with refractory epilepsy by improving seizure warning systems. However, further multi-center, prospective research is needed to validate these findings and to refine automatic detection algorithms for broader application. Specifically, improvements in automated artifact rejection and machine learning based classification could reduce the need for extensive visual confirmation of HFOs.

### 4.6 Limitations

Despite these promising findings, several limitations must be acknowledged. A key methodological consideration is the sampling frequency used in our study. To determine whether our sampling frequency (512 Hz) influenced the results, we performed a comparison with previous studies. The sampling frequency limited our analysis to HFOs within the ripple band (80–250 Hz). To assess whether this affected our findings, we compared the peak HFO frequencies from an age-matched subset of our patients (ages 3–9 years) with those reported in key pediatric scalp EEG studies. Our analysis yielded a mean peak frequency of 100.53 ± 16.06 Hz and a median of 95.20 [92.10–104.10] Hz, which aligns closely with values previously reported in the literature.

Our findings are notably consistent with those of Chaitanya et al. (2015), who observed a mean HFO frequency of 96.4 ± 11.4 Hz in children with absence epilepsy despite their significantly higher sampling rate of 2048 Hz. This similarity suggests that our sampling frequency did not introduce substantial bias or artificially lower the detected peak frequencies. Additionally, the peak frequencies identified in our cohort fall within the range 97.7–140.6 Hz (mean:119.5 Hz) reported by Kobayashi et al. (2010). Importantly, that study effectively characterized ripple band HFOs using scalp EEG recorded at a comparable sampling frequency of 500 Hz, further supporting the feasibility and validity of our methodological approach. While our chosen sampling frequency represents a potential limitation, this comparative analysis clearly demonstrates that our methodology was sufficient for the reliable detection and characterization of ripple band HFOs. Other limitations include the retrospective design of our study and the relatively small sample size, particularly for seizures recorded during sleep which may constrain the generalizability of our findings. Moreover, our semi-automated detection algorithm required visual verification, introducing an element of subjectivity. Variations in sampling frequency (512 Hz vs. 500 Hz in a few cases) and the exclusion of patients with significant artifacts further highlight the need for standardized recording protocols.

## 5 Conclusion

In summary, our study demonstrates that scalp EEG recordings at a 512 Hz sampling rate can effectively capture HFOs. The significant lateralization of HFO rates to the EH suggests their utility in noninvasively identifying the hemisphere responsible for seizure generation. Additionally, the observed increase in HFO rates during the pre-ictal period may support their potential role in predicting seizures. We observed an age-dependent decline in HFO rates, with younger patients exhibiting higher median values than older patients, consistent with findings reported in earlier studies. Although our results did not establish a strong connection between HFO rates and precise SOZ localization, they underscore the clinical relevance of scalp-recorded HFOs as potential biomarkers in the presurgical evaluation of patients with drug-resistant epilepsy.

## CRediT authorship contribution statement

**Dhruva P. Achar:** Conceptualization, Data curation, Investigation, Methodology, Software, Formal analysis, Visualization, Validation, Writing – original draft. **Karunakar A. Kotegar:** Conceptualization, Project administration, Resources, Supervision, Writing – review & editing. **Kurupath Radhakrishnan:** Conceptualization, Data curation, Resources, Investigation, Supervision, Validation, Writing – review & editing.

## Declaration of Generative AI and AI-assisted technologies in the writing process

During the preparation of this work, the authors used ChatGPT by OpenAI to improve grammar and readability. After using this tool, the authors reviewed and edited the content as needed and takes full responsibility for the content of the publication.

## Funding

No funding was received for this study.

## Declaration of competing interest

The authors declare that they have no known competing financial interests or personal relationships that could have appeared to influence the work reported in this paper.
